# When Conventional Oxygen Therapy Fails—The Effectiveness of High-Flow Nasal Oxygen Therapy in Patients with Respiratory Failure in the Course of COVID-19

**DOI:** 10.3390/jcm10204751

**Published:** 2021-10-16

**Authors:** Marta Rorat, Wojciech Szymański, Tomasz Jurek, Maciej Karczewski, Jakub Zelig, Krzysztof Simon

**Affiliations:** 1 Department of Forensic Medicine, Wroclaw Medical University, 50-367 Wroclaw, Poland; marta.rorat@gmail.com; 2 Department of Infectious Diseases and Hepatology, Wroclaw Medical University, 50-367 Wroclaw, Poland; szymanski.wojciech9@gmail.com (W.S.); krzysimon@gmail.com (K.S.); 3 Department of Applied Mathematics, Wroclaw University of Environmental and Life Sciences, 50-375 Wroclaw, Poland; maciej.karczewski@manteion.pl; 4 Second Infectious Diseases Ward, Gromkowski Regional Specialist Hospital in Wroclaw, 51-149 Wroclaw, Poland; jakub.zelig@gmail.com

**Keywords:** acute respiratory distress syndrome, SARS-CoV-2, oxygen therapy

## Abstract

High-flow nasal oxygen (HFNO) is recommended as a first-line treatment in patients with acute hypoxemic respiratory failure due to COVID-19. We assessed the effectiveness of HFNO and predictors of failure and death. The medical records of 200 consecutive adult patients treated with HFNO were analysed. Ninety-two patients (46%) were successfully cured, 52 (26%) required noninvasive ventilation, and 61 (30.5%) received intubation. Overall mortality was 40.5%. Risk factors of HFNO ineffectiveness were: SpO_2_ ≤ 90% with conventional oxygen therapy (HR 0.32, 95% CI 0.19–0.53, *p* < 0.001), SpO_2_ ≤ 74% without oxygen therapy (HR 0.44, 95% CI 0.27–0.71, *p* < 0.001), an age ≥ 60, comorbidities, biomarkers (C-reactive protein, procalcitonin, creatinine, lactate dehydrogenase), duration of symptoms before admission to hospital ≤ 9 days, start of treatment with HFNO ≤ 4 days. The multivariate logistic regression models (age ≥ 60, comorbidities, C-reactive protein concentration and SpO_2_ with oxygen therapy) revealed a high predictive value of death and HFNO failure (AUC 0.851, sensitivity 0.780, specificity 0.802; AUC 0.800, sensitivity 0.776, specificity 0.739, respectively). HFNO is a safe method for treating acute hypoxemic respiratory failure, with effectiveness reaching nearly 50%. Low values of SpO_2_ without and during oxygen therapy seem to be good diagnostic tools for predicting death and HFNO failure.

## 1. Introduction

The COVID-19 pandemic (coronavirus disease 2019) has resulted in doctors from various specialisations having to face multiple challenges not only in terms of organising a system for patient health care, implementing procedures with patients in complete isolation, interpreting results of new diagnostic tests but also applying new or rarely used methods for treatment. As critical patients made up approximately 3–5% of the total number of patients [1,2], there was not sufficient capacity for them all to be admitted to intensive care units (ICUs). Therefore, in multiple establishments, critical patients were taken care of by doctors from conservative treatment departments. They needed to learn how to treat patients with respiratory failure, from recognising the symptoms and reacting rapidly to deterioration in health to choosing and applying suitable treatment methods.

SARS-CoV-2 infection manifests itself initially as a mild flulike illness. However, in some cases, severe pneumonia and acute respiratory distress syndrome (ARDS) develop rapidly [3]. ARDS is characterized by acute respiratory distress (in which pulmonary edema cannot be fully explained by heart disease or fluid overload) associated with hypoxemia and the presence of bilateral infiltrate on chest imaging [4]. An acute and diffuse inflammatory damage into the alveolar-capillary barrier associated with an increase in vascular permeability, reduced compliance and the volume of the aerated lung tissue often occurs in ARDS. These disorders result in compromising gas exchange and hypoxemia, requiring oxygen support [3]. Next to conventional oxygen therapy and mechanical ventilation high-flow nasal oxygen (HFNO), which had been previously used in ICUs or in pulmonary departments, became the therapy of choice, recommended by multiple scientific societies in case of ineffectiveness of conventional oxygen therapy in patients with early acute hypoxemic respiratory failure due to COVID-19 [5,6,7,8,9].

A high-flow oxygen system provides oxygen-rich, appropriately heated and humidified gas to a patient’s nose at high velocity to deliver stable, precisely set high fraction of inspired oxygen (FiO_2_). Most HFNO device flow rates reach up to 60 L/min. HFNO reduces anatomical dead space, provides low levels of positive end-expiratory pressure (PEEP) (3 cm H_2_O on average), and decreases respiratory workload and breathing frequency. It has been proven that the use of HFNO compared to conventional oxygen therapy is associated with a lower risk of subsequent intubation, admission to an intensive care unit and mortality in hypoxemic respiratory failure. Also important is that HFNO is relatively easy to use and comfortable for a patient. However, it consumes large amounts of oxygen and requires a cooperative patient with an unobstructed nasal cavity [10,11,12,13,14].

The effectiveness of HFNO in patients with COVID-19 is still being researched. There are currently no defined criteria for HFNO implementation, continuation and failure. The aim of our work was to assess the effectiveness and to find risk factors of death and ineffectiveness of HFNO therapy in patients with hypoxemic respiratory failure in the course of COVID-19.

## 2. Materials and Methods

In a retrospective study, we analysed the medical records of 200 out of 248 consecutive adult patients with severe COVID-19, hospitalized from 01 September 2020 to 10 July 2021 on infectious diseases wards at Specialist Regional Hospital in Wroclaw (Poland). Inclusion criteria comprised:

1. Confirmed SARS-CoV-2 infection (positive RT PCR test, reverse transcription polymerase chain reaction).
2. Clinical features of critical disease during hospitalisation, according to the criteria provided by the Chinese Centre for Disease Control: adult respiratory distress syndrome (ARDS) or respiratory failure, septic shock, and/or multiple organ dysfunction (MOD) or failure (MOF) [1].
3. Acute hypoxemic respiratory failure requiring >15 L/min of oxygen and treated with high-flow nasal oxygen therapy due to hospitalisation.

Exclusion criteria comprised:

1. Death within 24 h of hospital admission.
2. Using HFNO for less than 24 h.
3. Patients who did not receive any lab tests.
4. Patients transferred from other hospitals in which HFNO, noninvasive ventilation (NIV) or respiratory therapy had been used.
5. Patients transferred from ICUs.
6. Patients transferred to other hospital departments because of complications in SARS-CoV-2 infection and/or its treatment, excluding pulmonary and ICU departments where respiratory failure treatment was continued.

Patients were assigned to HFNO by infectious diseases specialists. Doctors did not follow standardised criteria when implementing treatments. The decision to implement HFNO in each case was taken separately based on a patient’s condition and test results and assessment of the patient’s cooperation. High-flow nasal oxygen was delivered by an Airvo 2 (Fisher&Paykel healthcare, New Zealand) or HFNC device (Respircare, China).

The medical report comprised a patient’s medical history, duration of symptoms, results of computed tomography (CT) scan performed at the time of deterioration of respiratory capacity, oxygen saturation (SpO_2_) measured by pulse oximeter (on the day of administering HFNO; during oxygen therapy using a non-rebreather mask with maximum flow—15 L/min, and without oxygen therapy), partial pressure of oxygen (PO_2_) from capillary blood (taken on the day of administration of HFNO, during oxygen therapy using a non-rebreather mask with maximum flow), results of laboratory tests performed on admission as well as on the day of administering HFNO therapy: complete blood count, lactate dehydrogenase (LDH) activity, levels of C-reactive protein (CRP), procalcitonin (PCT), D-dimer, fibrinogen, creatinine and ferritin.

Chest CT examinations were performed using a 64-slice CT scanner with tube potential 120 kVp; detector configuration, 64 × 0.625 mm; rotation time 0.4 s (PHILIPS INGENUITY CORE 64). A semi-quantitative CT score was calculated based on the extent of lung involvement according to a modified version of the scale proposed by Pan and co-researchers: 1: <5%; 2: 5–25%; 3:26–50%; 4: 51–75%; 5, >75% [15].

HFNO failure was defined as the need for NIV or endotracheal intubation or death whilst under HFNO. The decision to use NIV or intubation was determined by experienced anaesthesiologists. When assessing the need to escalate therapy, the following factors were considered: comorbidities, SpO_2_, respiratory rate, respiratory effort, results of arterial blood gas analysis, mental state and circulatory efficiency.

Descriptive statistics of demographic data, risk factors and clinical data were presented as mean values with standard deviations, median with interquartile range, or number of cases with percentage. Survival analysis was assessed using the Kaplan–Meier survival curve. Comparison of survival curves was performed using a log-rank test. Analysis of the effect of different risk factors on risk of death or ineffectiveness of HFNO was performed using univariate and multivariate cox proportional hazard regression. Statistics presented for these models are hazards ratio and its 95% confidence interval. Model selection for multivariate models was performed using the Akaike Information Criterion (AIC). For quantitative variables, the optimal cutpoint was calculated using the Youden Index. ROC analysis was performed to assess the effectiveness of those cutpoints. Comparison of laboratory results on admission and on the day HFNO was started was performed using the Wilcoxon test for paired data. The R package for Windows (version 4.1, Microsoft Corporation, Albuquerque, NM, USA) was used for all calculations (statistical results were considered significant when the *p*-value was <0.05) [16].

## 3. Results

HFNO was ineffective in 108 out of 200 (54%) hospitalised patients. Data concerning the type and effectiveness of therapy applied in respiratory failure are shown in Table 1. In 22/108 patients (20.4%) therapy was not escalated because of sudden death, unfavourable prognosis (based on consultations with an anaesthetist) or lack of patient consent. NIV was administered in 52/108 (48.1%) of patients and 39/52 (75%) of these patients died. Mortality in the group of patients treated with NIV was statistically significantly higher (HR 2.20, 95% CI 1.42–3.42, *p* < 0.001) than when HFNO alone was used. 61/108 (56.5%) of patients were intubated, 45/61 (73.8%) of them died (Table 1). In this group of patients, mortality was statistically significantly higher (HR 2.39, 95% CI 1.53–3.73, *p* < 0.001) than when only HFNO was used. No correlation was found between late intubation and the risk of death (*p* = 0.454).

Independent variables with a significant negative impact on survival according to the cox proportional hazards regression model are presented in Table 2. The optimal cut-off was established for quantitative parameters. Area under the ROC curve (AUC), sensitivity and specificity for calculated cut points are shown in Table 3.

We did not prove that a longer duration of HFNO therapy using high flow parameters (≥60 L/min, FiO_2_ ≥0.8) was related to higher mortality. Similarly, no correlation was found between blood saturation without oxygen using maximum flow and extended time of HFNO therapy. The analysis performed revealed the opposite conclusion, which directly showed the ineffectiveness of this method and suggested the need to escalate the therapy. A negative correlation was found between the duration of HFNO therapy and the concentration of PCT (ρ = −0.33, *p* < 0.001), creatinine (ρ = −0.27, *p* < 0.001), activity of LDH (ρ = −0.21, *p* = 0.012), positive correlation with number of lymphocytes (ρ = 0.17, *p* = 0.021).

Multifactor analysis showed that hypertension is related to ischemic heart disease and is not an independent death risk factor.

Table 4 and Table 5 show the results of the analysis including chosen risk factors (including age ≥60 years old, presence of ischemic heart disease and chronic kidney disease, C-reactive protein concentration and SpO_2_ with or without oxygen therapy) of death and ineffectiveness of HFNO. Models with SpO_2_ without oxygen therapy showed higher specificity values, while SpO_2_ on oxygen therapy higher ROC AUC and sensitivity. Combining both variables in one model resulted in worse models with much lower specificity and insignificant SpO_2_ without therapy. This might be caused by the correlation between both parameters.

Using the Wilcoxon test for paired data, the authors compared lab tests results on admission to hospital and on the day of administering HFNO in patients in which the time between the two sets of results was at least 24 h—the results are shown in Table 6. A statistically significant decrease in creatinine and CRP was observed compared to the values on hospital admission, with a simultaneous increase in D-dimers and neutrophils; the LDH results were not relevant. The analysis had its limitations due to the small number of pairs of certain outcomes, which resulted from the retrospective character of the research and lack of standard medical procedures.

In the research group, all patients were treated with dexamethasone and enoxaparin, 193/200 (96.5%) were treated with antibiotics, 100/200 (50%) with convalescent plasma, 83/200 (41.5%) with remdesivir and 34/200 (17%) with tocilizumab. No correlation was found between the medications used and the efficiency of HFNO and survival rates.

In 18/200 (9%) patients hospitalized in the infectious diseases department, bacterial infections were diagnosed: three cases of sepsis (the sources of infection were respiratory tract, urinary tract and in one case unknown), 4 cases of secondary bacterial pneumonia, 14 urinary tract infections, including one patient with both bacterial pneumonia and urinary tract infection. Pulmonary embolism was diagnosed in 34/200 (17%) of patients. No correlation was found between bacterial infections or pulmonary embolism and death rate or HFNO ineffectiveness.

HFNO complications, precluding the continuation of treatment, were observed in six patients: five (2.5%) suffered from pneumothorax and epistaxis requiring tamponade was observed in one patient with hepatic cirrhosis.

## 4. Discussion

The current study results revealed that HFNO is an effective treatment option for patients with hypoxic respiratory failure, which is the main cause of death due to COVID-19. Patients in the critical condition make up approximately 3–5% of all patients with COVID-19. In this group, the fatality rate reaches 50%, whereas it does not usually exceed 5–10% among COVID-19 patients as a whole [17,18,19,20,21]. In our research group, mortality reached 40.5% (Table 7). Patients over 60–70 years old and with underlying diseases have higher fatality rates than younger patients without comorbidities [17,19,20,21]. Sex is not relevant in terms of morbidity, although some researchers showed male predominance among patients admitted to intensive care units and HFNO failure [22,23]. Even though in our cohort male patients were predominant, sex turned out not to be a factor determining mortality or effectiveness of HFNO, similar to the research by Calligaro et al. [24]. The most predominant chronic diseases were cardiovascular disease, obesity, malignancies, diabetes, chronic renal disease, chronic respiratory disease [1,17,19,20,23,25,26,27,28], which is consistent with our observations (Table 2).

Except age and comorbidities independently associated with higher risk of poor outcomes there are multiple biomarkers and results of image tests [29,30,31]. In our research, a significant association between death and ineffectiveness of HFNO was observed with respect to the values of CRP, procalcitonin, ferritin, D-dimers, creatinine, LDH and lymphocytes as well as intensification of changes in CT scans. We determined an optimal cutpoint for each parameter, which is shown in Table 2. Additionally, we used the Wilcoxon test for paired data to compare lab test results on the day of admission to hospital and on the day of HFNO administration (Table 3). During hospitalisation we detected a statistically significant decrease in CRP and creatinine concentration, and a simultaneous increase in D-dimers and neutrophils (Table 6). The results show that progress in respiratory failure is not caused only by progressing inflammation and that the degree of its progression is only significant up to a certain point. Additional unfavourable factors were: shorter duration of symptoms before admission to hospital and administration of HFNO, using HFNO for a short time, which may be associated with greater disease dynamics and progression of developing respiratory failure. Such a correlation was not observed by Hu et al. [23].

The high efficiency of HFNO had been proven in many studies before COVID-19. In a multicenter, open-label trial conducted in 310 patients with acute hypoxemic respiratory failure (PaO2/FiO_2_ ≤ 300 mm Hg), Frat et al. observed lower 90-day mortality in the HFNO group compared to the conventional oxygen therapy group (HR 2.01; 95% CI, 1.01–3.99) as well as in the NIV group (HR 2.50; 95% CI, 1.31–4.78). HFNO therapy in the group studied as a whole did not result in a significantly different intubation rate. However, positive correlation was found by the authors in the subgroup of patients with PaO_2_/FiO_2_ ≤200 mm Hg [11]. Meta-analysis of eight trials with 1084 patients demonstrated the superiority of HFNO over conventional oxygen therapy and NIV in reducing the rate of intubation (OR 0.48; 95% CI, 0.31–0.73) and ICU mortality (OR 0.36; 95% CI, 0.20–0.63) [31]. In the study by Messika et al. [32], in patients, who received HFNO as a first step for acute respiratory failure (with all stages of ARDS, mainly caused by pneumonia) treatment, the intubation rate was 40%. Fifty percent of mechanically-ventilated patients survived. Univariate analysis identified three features associated with intubation: SAPS II (Simplified Acute Physiology Score II), hemodynamic failure, and the lowest 12-h PaO_2_/FiO_2_. In turn, according to multivariate analysis, only SAPS II was significantly associated with an intubation requirement [32].

Based on this high effectiveness of HFNO therapy, before conducting reliable studies in patients with COVID-19, in worldwide and Polish recommendations we found information on the preferable use of this therapy in case of ineffectiveness of conventional oxygen therapy in patients with type-1 respiratory failure [7,8,33]. This therapy is currently used in patients with a high prognosis of survival as well as those not recommended for intubation, except those who are hemodynamically unstable, with abnormal mental status or multiorgan failure [6,7,8].

Our study shows that effectiveness of HFNO (understood as no requirement to escalate the therapy for respiratory failure, and discharge from hospital) in patients with severe respiratory failure is 46% (Table 7), which is in line with data in the literature—from 34 to 62% depending on the severity of ARDS [19,23,24,34,35,36]. In the group studied, all patients were primarily treated with HFNO. NIV and intubation (NIV over intubation) were only used in cases of inefficient HFNO or when it could not be continued for reasons attributable to a patient. Such a strategy was commonly used in multiple Polish hospitals and also used in Germany [37]. It is worth pointing out that there are currently no defined criteria for HFNO failure. Patients with hypoxemia (PaO_2_/FiO_2_ ≤ 150 mmHg), high respiratory rate and thoracoabdominal asynchrony without improvement with HFNO are potentially at high risk of HFNO failure and should be considered to receive mechanical ventilation [38,39].

According to the literature, the intubation rate in patients treated with HFNO ranges from 31 to 66% [19,24,34,35,36]; in our study group it reached 30.5% (61/200). Our relatively low rate of intubation is related to using NIV in some patients. By escalating the therapy with the use of NIV and/or intubation, we successfully cured 27 patients, which constitutes 13.5%. Finally, the mortality rate among HFNO failures was 75%, less than in the study by Calligaro et al. [24]. The effectiveness of intubation and NIV therapy differs significantly in the given studies, which results from when this treatment is determined, availability of beds with respirators, cooperation with a patient and recovery prognosis. The high heterogeneity of the population also prevents the drawing of conclusions. It is known, however, that delayed intubation increases ARDS mortality, so early recognition of ARDS severity and qualification to invasive mechanical ventilation is crucial for survival [40]. Mortality in intubated patients varies significantly depending on the country, from 25 to 97%, with the lowest percentage in Germany and the USA because of the high number of ICU beds [37].

Routine monitoring of blood saturation (SpO_2_) using pulse oximeter, respiratory rate, test results of arterial and capillary gasometry are used to monitor patients’ respiratory efficiency. In order to predict the risk of intubation in patients with pneumonia and acute respiratory failure treated with HFNO, it is recommended to use the respiratory rate oxygenation (ROX) index, measured by pulse oximetry/FiO_2_ to respiratory rate [41]. ROX index ≥4.88 after HFNO initiation indicates a low risk of intubation, and thereby use of HFNO might be continued, whereas ROX index <2.85 at 2 and <3.85 at 12 h after HFNC initiation is a predictor of HFNC failure. Values between 2.85 and 4.88 require strict monitoring and in each case individual verification of recommendations to escalate the treatment. The authors of the study pointed that SpO_2_/FiO_2_ has a greater weight than respiratory rate [41]. Tatkov S. explained that the ROX index is unlikely to fall below 4.88 if FiO_2_ does not exceed 0.5 regardless of the respiratory rate, but it would be below the cut-off point if FiO_2_ is ≥0.8 in patients with increased respiratory rate [42]. The clinical usefulness of the ROX index has also been confirmed in patients with COVID-19. According to Celejewska-Wójcik et al., ROX index <3.85 measured within the first 12 h of therapy was related to increased mortality (HR 5.86; 95% CI, 3.03–11.35) [19]. In the study by Hu et al. [23], the ROX index (>5.55), as well as SpO_2_/FiO_2_, PaO_2_/FiO_2_ after 6 h of HFNO treatment demonstrated good prediction accuracy (AUROC, 0.798, 0.786, 0.749, respectively).

In our study we focused on predicting the ineffectiveness of HFNO therapy before implementing it. To do this, we used two accessible parameters of respiratory effectiveness: SpO_2_ without oxygen and with maximum flow through a non-rebreather mask, and oxygen pressure (PO_2_) in capillary blood. Use of the ROX index was not possible because of a lack of routine monitoring of number of breaths in all patients together with difficulties in precisely defining FiO_2_ when using low-flow oxygen therapy. The above-mentioned restrictions resulted from a very high number of severely ill patients and a limited number of medical staff (real world study). We used the results of capillary blood tests because it was easy and safe to obtain the sample in almost all conditions; the sample is usually taken by a nurse or a paramedic. We showed that SpO_2_ with and without oxygen therapy results in independent variables with a significant negative impact on survival and HFNO effectiveness (*p* < 0.001). Such a correlation was not found for PO_2_ from capillary blood, which proves the limited impact of this parameter in patients with hypoxemic respiratory failure.

Ultimately, the recommended range of oxygen saturation in patients with COVID-19 without known chronic lung disease is 92–96% [6,9]. These values were assumed taking into account tests and examinations with critical illnesses, including ARDS, which showed that target SpO_2_ 88–92% as well as ≥96% are associated with an increased risk of death [33,43,44]. The above results are somewhat controversial as they are not based on the results of the selected group of patients with ARDS, and they do not refer to patients with COVID-19. According to some researchers it would be advisable to target an oxygen saturation at least at the upper end of the recommended range [45]. According to the WHO, adults requiring urgent treatment should receive oxygen therapy to target SpO_2_ ≥ 94%. In addition, for stable patients target values of SpO_2_ are >90% [7]. In the research group the cut-off point for SpO_2_ in passive oxygen therapy through a non-rebreather mask with maximum flow was established at 90%. A significant increase in the risk of death and ineffectiveness of high-flow oxygen therapy were observed at this value or lower. Similar calculations were made for the value of saturation without oxygen, establishing the cut-off point at 71% with respect to the risk of death and 74% regarding ineffectiveness of HFNO. In the study by Calligaro et al. [24], median SpO_2_ in the group of patients successfully treated with HFNO was 91%; in failures it was 89% (*p* < 0.001). Those results are hard to compare to ours as this parameter was measured during HFNO and not, like in our study, before administering HFNO. The multivariate analysis revealed that after adjustment for age, ischemic heart disease, chronic kidney disease, SpO_2_ during oxygen therapy and CRP when administering HFNO, the predictive value of death and ineffectiveness of HFNO therapy was high—AUC 0.851 and 0.800, respectively (Table 5).

## 5. Limitations and Future Direction for Researches

Our study has some limitations. Firstly, this is a retrospective study, without a control group. The group studied is quite small and not homogenous. The reason for the small number of patients is the fact that the study was done in a single medical centre and involved only selected wards, which were consistent in the management of patients with respiratory failure. Short time frame was an additional limitation—in the first months of the pandemics, our experience in the field of HFNO therapy was limited and only little medical data was collected. There was also no protocol of qualifying and unified rules of running of HFNO therapy. The decisions about transition to NIV or invasive mechanical ventilation were taken individually by attending anaesthetists. In some cases, clinical data was incomplete, which could affect the results of statistical analysis, particularly of the multivariate prediction model.

Despite the limitations, the study accentuates a range of risk factors for the ineffectiveness of high-flow oxygen therapy in patients with COVID-19 and respiratory failure, which are easy to determine in clinical practice. There is a need for further research aimed at identification of parameters determining the effectiveness of conventional oxygen therapy, HFNO and NIV. Discovering such factors will enable the implementation of solutions aimed at optimizing the therapy and its adaptation to individual patient’s needs. In the long term, research should focus on assessing the effectiveness of individual therapeutic interventions in order to maximize survival. Conducting prospective, multicenter studies on large groups of patients is necessary to obtain reliable data.

## 6. Conclusions

1. Given the limited access to ICU during the pandemic, HFNO is a safe and easy-to-use way of treating acute hypoxemic respiratory failure due to COVID-19, with effectiveness reaching nearly 50%. Unfortunately, mortality in patients in whom HFNO fails is high.
2. SpO2 ≤ 90% with conventional oxygen therapy and SpO2 ≤ 74% without oxygen therapy is a good diagnostic tool in predicting HFNO failure, especially when other risk factors coexist.
3. The other factors responsible for HFNO failure include being aged over 60, comorbidities (ischemic heart disease, chronic kidney disease, malignant neoplasm, obesity), several biomarkers (C-reactive protein, procalcitonin, creatinine, LDH), duration of symptoms before hospital admission ≤ 9 days and before administering HFNO therapy ≤ 4 days.

## Figures and Tables

**Table 1:** Patients ineffectively treated with HFNO.

*n* = 200	HFNO	HFNO + NIV	HFNO+ Intubation	HFNO + NIV + Intubation
*n* (%)	108 (54%)	25 (12.5%)	34 (17%)	27 (13.5%)
Death	81/108 (75%)	14/25 (56%)	20/34 (58.8%)	25/27 (92.6%)

**Table 2:** Univariate cox regression model for survival and HFNO effectiveness as dependent variables.

	Death				HFNO Ineffectiveness			
	Cut-Off	HR	95% CI	*p*-Value	Cut-Off	HR	95% CI	*p*-Value
Sex	-	0.94	0.59, 1.49	0.784		0.89	0.59, 1.33	0.573
Age (years)	>60	2.91	1.45, 5.82	0.003	>62	1.54	0.93, 2.53	0.094
Concomitant diseases								
Hypertension		1.79	1.08, 2.98	0.024		2.04	1.32, 3.16	0.001
Ischemic heart disease		2.81	1.77, 4.45	<0.001		2.54	1.67, 3.85	<0.001
Atrial fibrillation		1.53	0.83, 2.84	0.172		1.33	0.75, 2.33	0.328
Pulmonary diseases		0.53	0.19, 1.44	0.213		0.83	0.40, 1.71	0.619
Malignant neoplasm		2.6	1.37, 4.93	0.004		1.35	0.70, 2.62	0.374
Obesity		1.08	0.69, 1.67	0.746		1.48	1.01, 2.18	0.045
Diabetes		1.4	0.90, 2.20	0.137		1.29	0.87, 1.91	0.209
Chronic kidney disease		2.97	1.60, 5.51	<0.001		3.09	1.75, 5.46	<0.001
Autoimmune diseases		1.42	0.83, 2.43	0.201		1.36	0.86, 2.14	0.183
Duration of symptoms before admission to hospital (days)	≤7	0.47	0.30, 0.75	0.001	≤9	0.33	0.20, 0.52	<0.001
Duration of symptoms before administration of HFNO (days)	≤7	0.61	0.38, 0.97	0.039	≤4	0.09	0.04, 0.20	<0.001
Number of days with HFNO	≤6	0.27	0.15, 0.47	<0.001	≤4	0.09	0.04, 0.20	<0.001
CT score	5	2.98	1.35, 6.56	0.007	5	2.92	1.49, 5.76	0.002
SpO_2_ without oxygen therapy	≤71	0.37	0.22, 0.63	<0.001	≤74	0.44	0.27, 0.71	<0.001
SpO_2_ with oxygen therapy	≤90	0.32	0.17, 0.60	<0.001	≤90	0.32	0.19, 0.53	<0.001
PO_2_ from capillary vessels	≤60	0.82	0.48, 1.38	0.453	≤52	0.67	0.43, 1.03	0.065
Lab parameters on the day of administration of HFNO								
CRP, mg/L	≥93.81	1.75	1.06, 2.86	0.028	≥137.7	1.67	1.13,2.46	0.01
Procalcitonin, ng/mL	≥0.358	3.87	2.27, 6.61	<0.001	≥0.367	4.28	2.67, 6.87	<0.001
Ferritin, ng/mL	≥1672.1	2.71	1.52, 4.86	<0.001	≥1672.1	1.61	0.96, 2.69	0.07
D-dimer, ng/mL	≥989	1.85	1.05, 3.26	0.032	≥989	1.53	0.94, 2.48	0.086
Fibrinogen, g/L	≥7.27	1.79	0.71, 4.54	0.219	≥7.27	2.08	0.88, 4.93	0.096
Creatinine, mg/dL	≥1.13	3.7	2.33, 5.86	<0.001	≥1.13	3.37	2.25, 5.06	<0.001
Lymphocytes, ×10^3^/µL	≤0.7	0.55	0.34, 0.87	0.011	≤1.1	0.62	0.36, 1.06	0.078
Neutrophiles, ×10^3^/µL	≥7.9	1.37	0.86, 2.19	0.186	≥7.9	1.41	0.95, 2.11	0.092
Lymphocytes/neutrophils index	≤0.08	0.54	0.34, 0.86	0.01	≤0.055	0.52	0.33, 0.83	0.006
LDH, U/L	≥672	2.32	1.35, 3.97	0.002	≥671	2.3	1.43, 3.69	<0.001

**Table 3:** Area under the ROC curve (AUC), sensitivity and specificity for calculated cut points.

	Death				HFNO Ineffectiveness			
Effect of	Cut-Off	AUC	Sensitivity	Specificity	Cut-Off	AUC	Sensitivity	Specificity
Age (years)	>60	0.67	0.88	0.38	>62	0.64	0.8	0.42
Duration of symptoms before admission to hospital (days)	≤7	0.53	0.61	0.50	≤9	0.56	0.77	0.35
Duration of symptoms before administration of HFNO (days)	≤7	0.48	0.32	0.7	≤4	0.51	0.06	0.99
Number of days with HFNO	≤6	0.73	0.8	0.61	≤4	0.81	0.81	0.73
CT score	=5	0.66	0.59	0.72	5	0.68	0.57	0.76
SpO_2_ without oxygen therapy	≤71	0.67	0.65	0.73	≤74	0.68	0.69	0.66
SpO_2_ with oxygen therapy	≤90	0.7	0.85	0.47	≤90	0.71	0.82	0.52
PO_2_ from capillary vessels	≤60	0.52	0.65	0.45	≤52	0.56	0.41	0.78
CRP, mg/L	≥93.81	0.61	0.74	0,46	≥137.7	0.62	0.52	0.69
Procalcitonin, ng/mL	≥0.358	0.73	0.66	0.78	≥0.367	0.76	0.59	0.87
Ferritin, ng/mL	≥1672.1	0.62	0.54	0.73	≥1672.1	0.55	0.44	0.7
D-dimer, ng/mL	≥989	0.6	0.78	0.4	≥989	0.62	0.78	0.45
Fibrinogen, g/L	≥7.27	0.55	0.33	0.86	≥7.27	0.56	0.32	0.88
Creatinine, mg/dL	≥1.13	0.68	0.51	0.91	≥1.13	0.68	0.43	0.94
Lymphocytes, ×10^3^/µL	≤0.7	0.59	0.54	0.64	≤1.1	0.57	0.84	0.30
Neutrophiles, ×10^3^/µL	≥7.9	0.54	0.5	0.65	≥7.9	0.59	0.52	0.71
Lymphocytes/neutrophils index	≤0.08	0.59	0.44	0.73	≤0.055	0.61	0,27	0.91
LDH, U/L	≥672	0.63	0.41	0.86	≥671	0.63	0.4	0.9

**Table 4:** Analysis of effect of SpO_2_ without oxygen therapy on risk of death and HFNO inefficiency in a multifactor logistic regression model.

	Death			HFNO Inefficiency		
Effect of	HR	95% CI	*p*-Value	HR	95% CI	*p*-Value
Age ≥60	4.75	1.65, 13,7	0.004	1.36	0.69, 2.66	0.38
SpO_2_ without oxygen therapy	0.97	0.945, 0.995	0.019	0.97	0.95, 0.99	0.004
Ischemic heart disease	2.26	1.42, 4.84	0.002	1.77	0.997, 3.14	0.051
Chronic kidney disease	2.91	1.25, 6.76	0.013	3.61	1.63, 7.99	0.002
CRP, mg/L	1.004	1.001, 1.007	0.002	1.004	1.001, 1.006	0.003
AUC	0.825			0.784		
Sensitivity	0.661			0.553		
Specificity	0.861			0.899		

**Table 5:** Analysis of effect of SpO_2_ with oxygen therapy on risk of death and HFNO inefficiency in a multifactor logistic regression model.

	Death			HFNO Inefficiency		
Effect of	HR	95% CI	*p*-Value	HR	95% CI	*p*-Value
Age ≥60	3.75	1.31, 10.7	0.014	1.13	0.57, 2.23	0.73
SpO_2_ on oxygen therapy	0.90	0.85, 0.94	<0.001	0.92	0.89, 0.96	<0.001
Ischemic heart disease	3.42	1.92, 6.08	0.004	2.21	1.27, 3.84	0.005
Chronic kidney disease	1.96	0.83, 4.64	0.162	2.76	1.25, 6.11	0.012
CRP, mg/L	1.005	1.002, 1.007	<0.001	1.004	1.001, 1.006	0.001
AUC	0.851			0.800		
Sensitivity	0.780			0.776		
Specificity	0.802			0.739		

**Table 6:** Comparison of lab test results on hospital admission and on the day of administering HFNO, Wilcoxon test for paired data. Effect size was calculated using rank-biserial correlation.

Laboratory Parameters	Number of Pairs	On Admission, Median	I = When Implementing HFNO, Median	*p*-Value	Effect Size	CI 95%
CRP, mg/L	105	122.5	103.4 (↓)	0.014	−0.28	−0.4, −0.07
Procalcitonin, ng/mL	38	0.27	0.18	0.517	−0.12	−0.45, 0.24
Ferritin, ng/mL	16	1336.4	1071.7	0.518	−0.19	−0.64, 0.35
D-dimer, ng/mL	96	1075.5	1360 (↑)	0.007	0.32	0.10, 0.51
Creatinine, mg/dL	90	1.05	0.83 (↓)	<0.001	−0.82	−0.88, −0.72
Lymphocytes, ×10^3^/µL	83	0.80	0.90	0.404	−0.11	−0.34, 0.14
Neutrophiles, ×10^3^/µL	83	5.50	7.50 (↑)	<0.001	0.61	0.43, 074
LDH, U/L	51	485	523	0.056	0.31	2.61 × 10^−3^, 0.56

↓ decrease in lab test result on the day of administering HFNO compared to the value on hospital admission, ↑ increase decrease in lab test result on the day of administering HFNO compared to the value on hospital admission.

**Table 7:** Baseline demographics and clinical characteristics (*n* = 200).

Characteristic	Number of Patients
Sex	
Male	134 (67%)
Female	66 (33%)
Age	65.2 (13.1), 67.0 (60.0–74.0)
Concomitant diseases	
Hypertension	128 (64%)
Ischemic heart disease	38 (19%)
Atrial fibrillation	21 (10.5%)
Pulmonary diseases	15 (7.5%)
Malignant neoplasm	17 (8.5%)
Obesity	95 (47.5%)
Diabetes	68 (34%)
Chronic kidney disease	16 (8%)
Autoimmune diseases	20 (10%)
CT score	4; 2–5
SpO_2_ without oxygen therapy (%)	71.8 (11.0), 74 (68–80)
SpO_2_ with oxygen therapy (%)	88.3 (5.1), 89 (86–92)
PO_2_ in capillary blood (mm Hg)	60.7 (14.8), 58 (50–70)
Laboratory test results during administration of HFNO	
CRP (≤6 mg/L)	138.5 (92.6), 123.0 (63.2–200.7)
Procalcitonin (≤0.05 ng/mL)	1.04 (2.76), 0.24 (0.10–0.76)
Ferritin (≤291 ng/mL)	1958.7 (2099.5), 1337.2 (672.5–2517.4]
D-dimer (≤500 ng/mL)	3225.4 (5869.4), 1362.0 (877.0–2449.8)
Fibrinogen (≤3.5 g/L)	6.3 (1.5), 6.3 (5.0–7.2)
Creatinine (≤1.15 mg/dL)	1.14 (0.90), 0.89 (0.75–1.13)
Lymphocytes (≥1 × 10^3^/µL)	0.89 (0.44), 0.80 (0.60–1.10)
Neutrophiles (≥7 ×10^3^/µL)	8.1 (4.5), 7.4 (4.8–10.7)
Lymphocytes/neutrophils index	0.151 (0.156), 0.11 (0.07–0.20)
LDH (≤246 U/L)	602.6 (220.0), 553.0 (452.2–671.2)
Respiratory support	
Noninvasive ventilation	52 (26%)
Respiratory therapy	61 (30.5%)
Duration of HFNO therapy (days)	6.8 (5.2), 5.5 (3.0–9.0)
HFNO flow ≥60 L/min and FiO_2_ ≥0.8 (days) (75.5%)	4.4 (3.5), 4.0 (2.0–6.0)
HFNO flow ≥40 L/min with FiO_2_ ≥0.6 (days) (89%)	5.9 (4.2), 5.0 (3.0–8.0)
Duration of symptoms before hospital admission	8.3 (4.1), 7.0 (6.0–10.0)
Duration of symptoms until administration of HFNO therapy	10.4 (4.9), 9.5 (7.0–13.0)
Duration of hospitalisation (days)	20.2 (15.6), 17.0 (12.0–25.0)
Death	81 (40.5%)
Duration of the disease before death (days)	28.4 (16.0), 25.0 (19.0–33.2)

Qualitative variables presented as counts and frequencies, quantitative variables presented as mean (SD), median (interquartile range).

## Data Availability

All relevant data are within the manuscript.
